# Renal Biomarkers for Treatment Effect of Ranibizumab for Diabetic Macular Edema

**DOI:** 10.1155/2020/7239570

**Published:** 2020-08-18

**Authors:** Ivan Pochou Lai, Wei-Lun Huang, Chung-May Yang, Chang-Hao Yang, Tzyy-Chang Ho, Yi-Ting Hsieh

**Affiliations:** ^1^National Taiwan University Hospital, Taipei, Taiwan; ^2^Department of Ophthalmology, National Taiwan University Hospital, Taipei, Taiwan; ^3^College of Medicine, National Taiwan University, Taipei, Taiwan

## Abstract

**Aims:**

To investigate the correlations between renal biomarkers and the treatment outcomes of ranibizumab for diabetic macular edema (DME).

**Methods:**

This hospital-based study retrospectively enrolled 88 eyes from 67 patients who had received one-year intravitreal ranibizumab treatment for DME. Best-corrected visual acuity (BCVA) and optical coherence tomography (OCT) at baseline and during the follow-up period were recorded. BCVA and OCT characteristics at baseline and their changes after ranibizumab treatment were compared between different proteinuria and estimated glomerular filtration rate (eGFR) groups.

**Results:**

Of the 88 eyes studied, those with moderately increased proteinuria had a thicker central subfield foveal thickness (CFT) and a higher proportion of intraretinal cysts than those with no proteinuria (*P* = 0.012 and 0.045, respectively) at baseline. After one year of ranibizumab treatment, the reduction in CFT was greater in patients with severely increased proteinuria than those with normal to mildly increased proteinuria (*P* = 0.030). On the other hand, patients with an eGFR <30 tended to have poorer visual improvements than those with normal eGFR (*P* = 0.044).

**Conclusions:**

After ranibizumab treatment for DME, patients with severe proteinuria tended to gain better anatomical improvement, while those with poor eGFR tended to have poorer visual improvement.

## 1. Introduction

Diabetic macular edema (DME) is the main reason for the visual deterioration in patients with diabetes [1]. With the advent of optical coherence tomography (OCT), investigators have classified the morphological patterns into diffuse retinal thickening, intraretinal cyst, and subretinal fluid [2]. The disorganization of the blood-retinal barrier was regarded as a key event in the development of DME. This process encompasses a wide variety of cytokines under chronic hyperglycemia, among which hypoxia-induced release of vascular endothelial growth factor (VEGF) played an essential role [3]. Ranibizumab (Lucentis; Genentech, South San Francisco, CA) is a humanized monoclonal antibody Fab fragment against all isoforms of VEGF-A. Intravitreal ranibizumab (IVR) have shown anatomical and visual improvements in several large randomized clinical trials (RCTs) [4, 5]. In light of these discoveries, anti-VEGF drugs have been the treatment of choice for DME in recent years [3].

Recently, researchers have shown an increasing interest in the association of DME and chronic kidney disease (CKD). Some cross-sectional studies revealed that macroalbuminuria, but not eGFR, was related to DME [6–8]. In a prospective cohort study conducted by our group [9], patients with DME at baseline also had higher serum creatinine and lower eGFR at baseline; but for those without DME, abnormal baseline urinary albumin/creatinine ratio (UACR), not eGFR, was significantly associated with the development of new-onset DME during the follow-up period. This implies that proteinuria and eGFR may play different roles in the pathophysiology of DME.

Given the growing studies concerning the association of CKD and DME, far too little attention has been paid to the impact of renal profiles on the treatment effect for DME. In the post hoc analysis of 2 randomized trials, RISE and RIDE, serum creatinine or eGFR was irrelevant to visual change after 2 years of ranibizumab treatment [10]. However, a retrospective study reported that patients with higher serum creatinine level were prone to poorer visual improvement following bevacizumab treatment for DME [11]. Despite the presumably distinct effects of proteinuria and eGFR as proposed above, no previous studies have dealt with the role of UACR in the treatment for DME. In this study, we aim to evaluate the association between renal biomarkers and the treatment outcomes of intravitreal ranibizumab for DME.

## 2. Materials and Methods

### 2.1. Study Population

This study retrospectively collected data from patients who started receiving IVR for DME at the National Taiwan University Hospital between January 2013 and December 2017. Inclusion criteria included the following: (1) diabetic retinopathy documented by fundus photography or fluorescein angiography (FA); (2) macular edema with the presence of retinal thickening, intraretinal cysts, or subretinal fluid and a central subfield foveal thickness (CFT) greater than 300 *μ*m as documented by optical coherence tomography (OCT); (3) best-corrected visual acuity (BCVA) between 20/400 and 20/40 at baseline; (4) available record of estimated glomerular filtration rate (eGFR) at baseline or during the first year of treatment; and (5) available record of UACR, urinary protein-creatinine ratio (UPCR), hemodialysis, or peritoneal dialysis at baseline or during the first year of treatment. Exclusion criteria included the following: (1) eyes with vitreomacular traction or tractional retinal detachment with or without macular involvement demonstrated by OCT or fundoscopy; (2) eyes with choroidal neovascularization or any other retinal vascular diseases such as retinal vein occlusion documented by FA; and (3) eyes that did not receive regular treatment and follow-up during the first year of treatment. After recruitment, a total of 88 eyes from 67 patients were enrolled in this study. All cases received three consecutive, monthly intravitreal injections of ranibizumab as the loading treatment, and then received treatment as needed after month 3 based on the combination of clinical presentation, doctors' suggestions, and patients' decision. Generally, if the macular edema had been subsided, or the BCVA and CFT had been stationary for two consecutive visits, no injection would be given. If recurrent macular edema was noted during the follow-up visits, ranibizumab injection would be given again. The subsequent follow-up interval was on a monthly basis and could be extended to up to 3 months given stable treatment outcome. This research adhered to the tenets of the Declaration of Helsinki, and approval was obtained from the Institutional Review Board of the National Taiwan University Hospital.

### 2.2. Data Collection

The following data was collected for all cases: best-corrected visual acuity (BCVA) and OCT measured at baseline, 3 months, 6 months, and 12 months. BCVA was measured with Snellen charts and was converted to the logarithm of the minimum angle of resolution (logMAR). CFT and the presence of intraretinal cysts or subretinal fluid at central fovea were obtained from OCT. Age, sex, status of hypertension, serum HbA1c level, and staging of diabetic retinopathy at baseline were recorded. Serum creatinine, UACR, and UPCR at baseline and during the follow-up periods were also recorded.

### 2.3. Measurements for Proteinuria and eGFR

The extent of proteinuria was classified into four groups: normal to mildly increased proteinuria, moderately increased proteinuria, severely increased proteinuria and dialysis. Those who had received hemodialysis or peritoneal dialysis before or during the study period belonged to the group of dialysis. Severely increased proteinuria was defined as having a UACR more than 0.3 or a UPCR more than 0.5 at baseline or any point in time during the first year of treatment. Moderately increased proteinuria was defined as having a UACR more than 0.03 or a UPCR more than 0.15, but no severely increased proteinuria at baseline or any time point during the first year of treatment. Those who always had a UACR less than 0.03 or a UPCR less than 0.15 were thought to have normal to mildly increased proteinuria. The eGFR was calculated using the equation recommended by the Chronic Kidney Disease Epidemiology Collaboration (CKD-EPI). The eGFR levels were divided into four groups for regression analysis: >90 mL/min, 61–90 mL/min, 30–60 mL/min, and <30 mL/min.

### 2.4. Statistical Analysis

BCVA was converted to the logarithm of the minimum angle of resolution (logMAR) for calculation. Wilcoxon rank-sum tests were used to compare the continuous variables, and Fisher's exact tests were used to examine the categorical variables between different groups of proteinuria and eGFR. Paired *t*-tests were used to compare the logMAR and CFT before and after treatment. Multiple linear or logistic regression models were used to evaluate the correlations between changes of BCVA or OCT characteristics and proteinuria or eGFR. When evaluating the effect of proteinuria, eGFR was adjusted in the regression models, and vice versa. Other covariates including age, hypertension, serum HbA1c level, DR staging, panretinal photocoagulation, baseline BCVA or OCT characteristics, and total injection numbers were also adjusted in the regression models. Stepwise covariate selection was used for all models to avoid over parametrization. A *P* value less than 0.05 was considered statistically significant. SAS 9.4 (SAS Institute Inc., Cary, NC, USA) was used for all statistical analyses.

## 3. Results

### 3.1. Baseline Characteristics

The mean age of the 65 patients was 62.0 ± 10.1 years (32 to 83 years); 33 were female and 32 were male. Seventy- four percent of them had hypertension, and the mean serum HbA1c level was 7.2 ± 1.1%. Of the 86 eyes studied, 2 had mild nonproliferative diabetic retinopathy (NPDR), 12 had moderate NPDR, 24 had severe NPDR, 15 had treatment-naive proliferative diabetic retinopathy (PDR), and 33 had PDR with previous panretinal photocoagulation. The mean logMAR of BCVA was 0.78 ± 0.38, and the mean CFT was 430 ± 112 *μ*m at baseline.  Table 1 showed the baseline characteristics in different proteinuria group. Eyes with moderately increased proteinuria had a thicker mean CFT (469 ± 96 *μ*m) than those with normal to mildly increased proteinuria (383 ± 73 *μ*m, *P* = 0.025).  Table 2 showed the baseline characteristics in different eGFR group. There were no differences in baseline BCVA or CFT among different eGFR groups (*P* > 0.05 for all).

### 3.2. Visual and Anatomical Improvements after Ranibizumab Treatment

After the ranibizumab treatment, the mean logMAR of BCVA improved from 0.78 ± 0.38 at baseline to 0.64 ± 0.36, 0.62 ± 0.34, and 0.63 ± 0.38 at months 3, 6, and 12, respectively (*P* < 0.001 for all). The mean CFT decreased from 430 ± 112 *μ*m at baseline to 308 ± 90 *μ*m, 323 ± 99 *μ*m, and 302 ± 93 *μ*m at months 3, 6, and 12, respectively (*P* < 0.001 for all). The mean injection number during the first year of ranibizumab treatment was 5.0 ± 2.2.

### 3.3. Correlations between Proteinuria and the Treatment Effects of Ranibizumab

**Figure 1:**
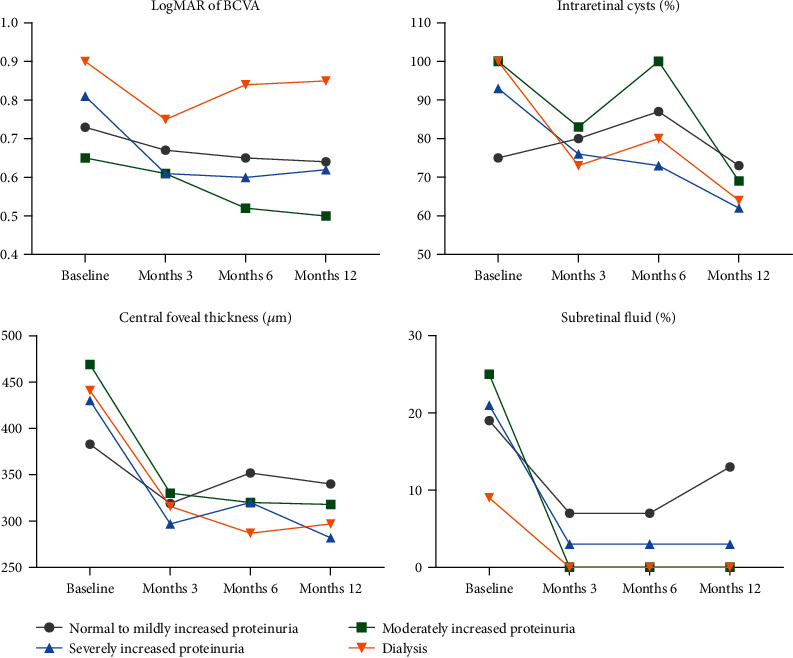
Changes in visual acuity (presented in logMAR of BCVA), central foveal thickness, presence of intraretinal cyst, and presence of subretinal fluid after intravitreal ranibizumab injection for diabetic macular edema in different groups of proteinuria.

Compared to eyes with normal to mildly increased proteinuria (3.8 ± 2.1), those with moderately or severely increased proteinuria received more ranibizumab injections within 12 months (6.0 ± 2.1, *P* = 0.008; and 5.4 ± 2.2, *P* = 0.015, respectively).  Figure 1 showed the changes in BCVA and OCT characteristics after ranibizumab use in four different proteinuria groups. No obvious differences in visual improvement were noted among the four groups. Those with proteinuria or under dialysis had thicker CFTs at baseline; however, they responded well to ranibizumab, and the CFTs became thinner than those without proteinuria at month 12 after the ranibizumab treatment. Similarly, those with proteinuria or under dialysis had higher proportions of intraretinal cysts at baseline, but they responded well to ranibizumab treatment with obvious resolution of intraretinal cysts. On the contrary, the proportion of intraretinal cyst did not decrease after ranibizumab treatment in those with normal to mildly increased proteinuria. As for subretinal fluid, the proportions were similar among the four groups at baseline; however, only those with normal to mildly increased proteinuria responded poorly to ranibizumab treatment in subretinal fluid resolution. After adjustment for baseline characteristics and injection numbers in multiple regression models, the reduction in CFT was still 69 *μ*m greater in eyes with severely increased proteinuria than those with normal to mildly increased proteinuria (*P* = 0.016) (Table 3).

### 3.4. Correlations between eGFR and Treatment Effects of Ranibizumab

**Figure 2:**
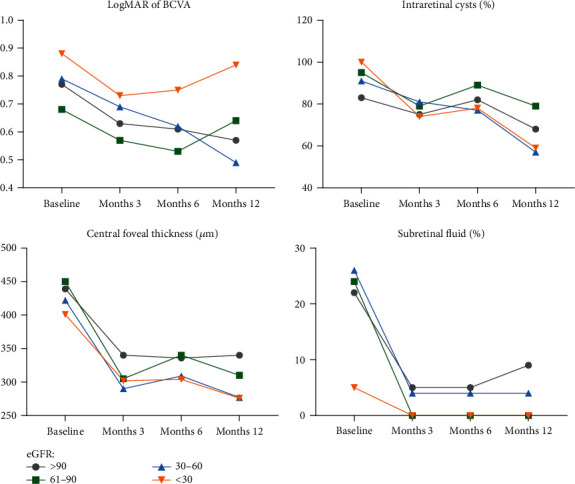
Changes in visual acuity (presented in logMAR of BCVA), central foveal thickness, presence of intraretinal cyst, and presence of subretinal fluid after intravitreal ranibizumab injection for diabetic macular edema in different groups of eGFR level.

Compared to eyes with an eGFR >90 (5.7 ± 2.2), those with an eGFR between 61 and 90 received less ranibizumab injections with 12 months (4.0 ± 2.3, *P* = 0.018).  Figure 2 showed the changes in BCVA and OCT characteristics after ranibizumab use in four different eGFR groups. Those with an eGFR <30 tended to have poorer baseline and final BCVA. No obvious trends in changes of CFT were noted among the four groups. The proportion of intraretinal cysts seemed to be higher in those with poor eGFR at baseline (although of no statistical significance), but the final results after treatment varied. As for subretinal fluid, those with normal eGFR seemed to respond worse to ranibizumab treatment in subretinal fluid resolution. After adjustment for baseline characteristics and injection numbers in multiple regression models, although those with an eGFR <30 had a borderline tendency of more reduction in CFT (*P* = 0.056), they still had poorer visual improvement when compared with those with an eGFR >90 (*P* = 0.040) (Table 4).

## 4. Discussion

In this study, we found that the severity of proteinuria was correlated with baseline CFT and the presence of intraretinal cysts at baseline. The association between albuminuria and DME had been reported in the previous studies [7–9, 12]. Both microalbuminuria and macroalbuminuria were recognized as risk factors for the development of DME, with the latter exerting a greater impact [6, 8]. Similar to our study, investigators have found no significant effects of eGFR on the baseline characteristics of DME [7–9, 13]. While albuminuria and eGFR were the two most important markers of diabetic CKD progression [14], distinct associations of DME with albuminuria and with eGFR might suggest different pathophysiological processes.

The current opinion on the development of DME has been largely focused on the breakdown of retinal barrier mediated by VEGF and other inflammatory cytokines [3, 15, 16]. Patients with CKD were found to have elevated serum VEGF, to which the eGFR level was inversely related [17]. Meanwhile, increased VEGF could also lead to higher vessel permeability and protein filtration in glomeruli [18] and in retina [3]. Nonsignificant associations between eGFR and DME, however, defied the pathogenesis of DME with the detrimental effects of VEGF alone. Admittedly, VEGF was a key to barrier dysfunction, but altered capillary dynamics controlled by the Starling forces could even compound the extent of fluid leakage upon barrier disruption [19]. Albuminuria with marked protein loss may lower the oncotic pressure and thus drove intravascular fluid into the interstitial tissue [20]. On the other hand, it was reported that overhydration in CKD was associated with DME [21]. The resolution of DME with systemic furosemide treatment, which was intended for volume expansion, was also observed in a few cases [22, 23]. Furthermore, proteinuria was an independent predictor for overhydration in CKD [24]. Intraglomerular hydrostatic pressure was proved to be correlated with urinary albumin excretion [25]. Considering these factors together, we hypothesized that proteinuria and DME may both result from increased hydrostatic pressure and were thus associated with each other.

**Figure 3:**
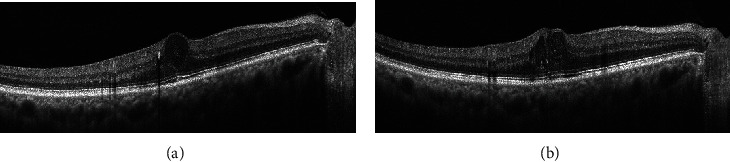
A 74-year-old male had an UACR of 0.029. (a) At baseline, there was cystic edema involving fovea in the right eye. (b) At month 12, the cystic edema still persisted after 8 ranibizumab injections were given.

Interestingly, though bearing a worse baseline condition, patients with more severe proteinuria showed better anatomical improvement after ranibizumab treatment for DME. Those with moderately to severely increased proteinuria even had thinner CFTs than those with normal to mildly increased proteinuria after ranibizumab treatment. As far as we know, this is a novel finding that has never been reported before. The mechanism underlying the formation of DME may furnish some clue to our observation. Macular edema was a consequence of fluid imbalance, which could be accredited to increased fluid entry, decreased drainage function, or the combination of both. VEGF held a global effect on retinal vascular endothelial and pericytes, including alteration of barrier junctional integrity, promotion of leukostasis, and increase in transcellular permeability [19]. Anti-VEGF therapy aimed at blocking the abovementioned processes, however, seemed unable to drain the fluid per se. Absorption of the edema relied on passive diffusion and the pumping function of retinal pigment epithelium and retinal Müller cells, which would be disturbed in streptozotocin-induced diabetic rat model [26–28]. On the other hand, patients with more severe proteinuria, as discussed in the previous section, may possess lower intravascular oncotic pressure or higher hydrostatic pressure, thus resulting in thicker CFTs. However, once the VEGF pathway was blocked by ranibizumab, the synergistic effect from the Starling forces would also diminish, ending up with unremarkable CFTs compared to those with normal to mildly increased proteinuria. We inferred that those with normal to mildly increased proteinuria were involved more in pumping dysfunction or inflammatory reaction than those with proteinuria did, thus leaving thicker CFTs after treatment.  Figure 3 showed an example of a case with normal to mildly increased proteinuria. The eye received 8 ranibizumab injections within 12 months, while the macular edema did not improve.

**Figure 4:**
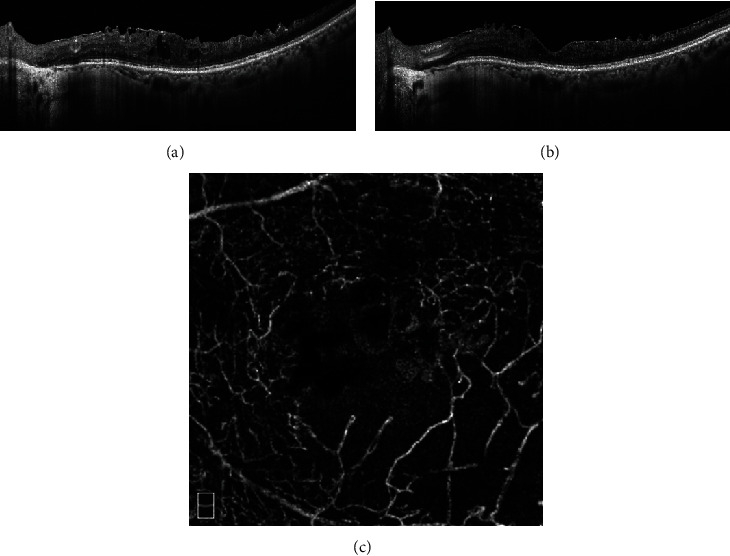
A 55-year-old male had an eGFR of 3.8. (a) At baseline, there was macular edema with intraretinal cysts involving fovea in the left eye. (b) At month 12, the macular edema improved a lot after 6 ranibizumab injections were given, while the vision did not improve. (c) OCTA showed severe ischemia at parafoveal areas.

It was also worth noting that patients with an eGFR <30 tended to gain poorer visual improvements despite comparable CFT reduction to the other groups. In a population-based study [29], it is found that retinal perfusion density in both superficial and deep layers decreased in subjects with lower eGFR. In diabetic patients, lower eGFR was also demonstrated to be independently associated with decreased retinal blood flow [30]. Nephropathy with either albuminuria or increased serum creatinine was associated with the presence of macular ischemia in eyes with diabetic retinopathy [31]. According to these results, we proposed that retinal perfusion may decline with the deterioration of eGFR, contributing to visual impairment caused by macular ischemia. Previous studies on OCT angiography (OCTA) can provide some hint about this. Although the vessel density in both the superficial and deep capillary plexus, which was lower in patients with DME, could improve after anti-VEGF therapy [32, 33], our study group reported that lower parafoveal vessel density in the superficial layers was associated with poorer visual improvement after adjustment for baseline BCVA and CFT [32]. Larger foveal avascular zone was also shown to be correlated with poorer VA in patients with resolved DME [34]. These suggest that besides macular edema, macular ischemia itself may contribute to visual impairment in cases with low eGFR, which results in the discrepancy between the anatomical improvement and visual improvement after ranibizumab treatment. However, only few cases in this study had received OCTA examinations, so that we could not evaluate the correlation between macular ischemia and eGFR.  Figure 4 showed an example of a case with an eGFR <30. After receiving 6 ranibizumab injections within 12 months, the macular edema improved a lot, while the vision did not improve due to severe macular ischemia as shown in OCTA.

Injection number could also affect the treatment outcomes. In this study, patients with moderately and severely increased proteinuria tended to receive more intravitreal injections. Treated with a PRN strategy after the loading phase, patients with frequent recurrence were supposed to receive more additional doses. Under the consideration of Starling forces as discussed previously, patients with proteinuria, even under a similar barrier condition to those without proteinuria, may suffer from edema recurrence in a shorter period due to hydrostatic or oncotic pressure. On the other hand, some patients might receive less-than-needed injections due to personal reasons or the reimbursement restriction. Therefore, the injection number was adjusted in all regression models to adjust for its effect on treatment outcomes.

The major limitation of this study was its retrospective design. We had no information about the duration and medication of diabetes, the body fluid status, or the duration and previous management for DME. In addition, our study population contained only 86 eyes from a single center, and thus, there was some inevitably inherent bias. Notwithstanding these limitations, this work offers valuable insights into the effects of renal profile on the treatment for DME. To the best of our knowledge, this is the first study to underscore the impact of renal function on the treatment effects of anti-VEGF agents for DME. Further prospective studies and randomized clinical trials are necessary for the determination of their causal relationships.

In conclusion, proteinuria rather than eGFR was associated with central retinal thickness and the presence of intraretinal cysts in DME patients at baseline. After ranibizumab treatment, those with severe proteinuria tended to gain better anatomical improvement, while those with poorer eGFR tended to have poorer visual improvement. These findings suggest a role of renal biomarkers for the evaluation of anti-VEGF treatment response for DME.

## Figures and Tables

**Table 1:** Demographic data, visual acuity, optical coherence tomography characteristics, and injection number in four different proteinuria groups at baseline.

	A: normal to mildly increased proteinuria (*n* = 16)	B: moderately increased proteinuria (*n* = 16)	C: severely increased proteinuria (*n* = 43)	D: dialysis (*n* = 11)	*P* value		
					A vs. B	A vs. C	A vs. D
Age (year)	63.2 ± 9.3	60.8 ± 5.4	64.4 ± 11.3	57.5 ± 9.8	0.41	0.74	0.13
Sex (female)	44%	69%	63%	18%	0.29	0.24	0.23
Hypertension	56%	56%	88%	73%	1	0.011	0.44
HbA1c (%)	7.7 ± 1.4	7.2 ± 0.8	7.2 ± 0.9	6.5 ± 0.8	0.50	0.45	0.027
DR staging					0.13	0.23	0.75
Mild NPDR	6%	0%	0%	9%			
Moderate NPDR	25%	6%	14%	9%			
Severe NPDR	31%	19%	30%	27%			
Treatment-naive PDR	19%	19%	16%	18%			
PDR with PRP	19%	56%	40%	36%			
LogMAR of BCVA	0.73 ± 0.36	0.65 ± 0.29	0.81 ± 0.42	0.90 ± 0.37	0.84	0.56	0.26
CFT (*μ*m)	383 ± 73	469 ± 96	430 ± 127	440 ± 103	0.025	0.49	0.19
Intraretinal cysts	75%	100%	93%	100%	0.10	0.078	0.12
Subretinal fluid	19%	25%	21%	9%	1	1	0.63
Injection number	3.8 ± 2.1	6.0 ± 2.1	5.4 ± 2.2	4.0 ± 1.5	0.008	0.015	0.41

CFT: central foveal thickness; DR: diabetic retinopathy; eGFR: estimated glomerular filtration rate; LogMAR of BCVA: logarithm of the minimum angle of resolution of best-corrected visual acuity; NPDR: nonproliferative diabetic retinopathy; PDR: proliferative diabetic retinopathy; PRP: panretinal photocoagulation.

**Table 2:** Demographic data, visual acuity, and optical coherence tomography characteristics in four different eGFR groups at baseline.

	A: eGFR >90 (*n* = 23)	B: eGFR 61-90 (*n* = 21)	C: eGFR 30-60 (*n* = 23)	D: eGFR <30(*n* = 19)	*P* value		
					A vs. B	A vs. C	A vs. D
Age (year)	59.0 ± 10.2	61.2 ± 8.0	69.2 ± 8.7	60.5 ± 10.2	0.84	0.002	0.50
Sex (female)	61%	67%	48%	42%	0.33	0.37	0.20
Hypertension	65%	57%	100%	74%	0.76	0.004	0.74
HbA1c (%)	7.5 ± 1.2	7.2 ± 0.9	7.5 ± 1.1	6.6 ± 0.8	0.57	0.77	0.030
DR staging					0.56	0.51	0.92
Mild NPDR	0%	0%	4%	5%			
Moderate NPDR	17%	10%	17%	11%			
Severe NPDR	26%	19%	39%	26%			
Treatment-naive PDR	17%	10%	22%	21%			
PDR with PRP	39%	62%	17%	37%			
LogMAR of BCVA	0.76 ± 0.35	0.68 ± 0.42	0.79 ± 0.42	0.88 ± 0.37	0.17	0.97	0.35
CFT (*μ*m)	439 ± 116	450 ± 128	423 ± 110	407 ± 93	0.99	0.50	0.54
Intraretinal cysts	83%	95%	91%	100%	0.35	0.67	0.11
Subretinal fluid	22%	24%	26%	5%	1	1	0.20
Injection number	5.7 ± 2.2	4.0 ± 2.3	5.7 ± 2.1	4.7 ± 1.8	0.018	0.95	0.18

CFT: central foveal thickness; DR: diabetic retinopathy; eGFR: estimated glomerular filtration rate; LogMAR of BCVA: logarithm of the minimum angle of resolution of best-corrected visual acuity; NPDR: nonproliferative diabetic retinopathy; PDR: proliferative diabetic retinopathy; PRP: panretinal photocoagulation.

**Table 3:** Correlation between proteinuria status and changes in visual acuity and optical coherence tomography characteristics after month 12 after ranibizumab treatment.

	Correlation coefficient or odds ratio				*P* value		
Changes at month 12	A: normal to mildly increased proteinuria (*n* = 16)	B: moderately increased proteinuria (*n* = 16)	C: severely increased proteinuria (*n* = 43)	D: dialysis (*n* = 11)	A vs. B	A vs. C	A vs. D
LogMAR of BCVA	Reference	-0.26	-0.21	-0.21	0.053	0.063	0.28
CFT (*μ*m)	Reference	-39	-69	-56	0.24	0.016	0.15
Intraretinal cysts	Reference	3.27	4.47	4.62	0.28	0.079	0.17
Subretinal fluid	Reference	^∗^	^∗^	^∗^	^∗^	^∗^	^∗^

CFT: central foveal thickness; LogMAR of BCVA: logarithm of the minimum angle of resolution of best-corrected visual acuity.

**Table 4:** Correlation between estimated glomerular filtration rate and changes in visual acuity and optical coherence tomography characteristics after Month 12 after ranibizumab treatment.

	Correlation coefficient or odds ratio				*P* value		
Changes at month 12	A: eGFR >90 (*n* = 23)	B: eGFR 61-90 (*n* = 21)	C: eGFR 30-60 (*n* = 23)	D: eGFR <30 (*n* = 19)	A vs. B	A vs. C	A vs. D
LogMAR of BCVA	Reference	0.115	-0.04	0.23	0.19	0.67	0.040
CFT (*μ*m)	Reference	-29	-50	-58	0.31	0.080	0.056
Intraretinal cysts	Reference	1.10	2..81	3.88	0.91	0.20	0.11
Subretinal fluid	Reference	^∗^	^∗^	^∗^	^∗^	^∗^	^∗^

CFT: central foveal thickness; eGFR: estimated glomerular filtration rate; LogMAR of BCVA: logarithm of the minimum angle of resolution of best-corrected visual acuity.

## Data Availability

Dataset will be available under request.
